# Skimmed Goat’s Milk Powder Enriched with Grape Pomace Seed Extract: Phenolics and Protein Characterization and Antioxidant Properties

**DOI:** 10.3390/biom11070965

**Published:** 2021-06-30

**Authors:** Danijel D. Milinčić, Aleksandar Ž. Kostić, Uroš M. Gašić, Steva Lević, Slađana P. Stanojević, Miroljub B. Barać, Živoslav Lj. Tešić, Viktor Nedović, Mirjana B. Pešić

**Affiliations:** 1 Faculty of Agriculture, Institute of Food Technology and Biochemistry, University of Belgrade, Nemanjina 6, 11080 Belgrade, Serbia; danijel.milincic@agrif.bg.ac.rs (D.D.M.); akostic@agrif.bg.ac.rs (A.Ž.K.); slevic@agrif.bg.ac.rs (S.L.); sladjas@agrif.bg.ac.rs (S.P.S.); baracm@agrif.bg.ac.rs (M.B.B.); vnedovic@agrif.bg.ac.rs (V.N.); 2 Department of Plant Physiology, Institute for Biological Research “Siniša Stanković”, National Institute of Republic of Serbia, University of Belgrade, Bulevar Despota Stefana 142, 11060 Belgrade, Serbia; uros.gasic@ibiss.bg.ac.rs; 3 Chair of Analytical Chemistry, Faculty of Chemistry, University of Belgrade, Studentski Trg 12–16, 11000 Belgrade, Serbia; ztesic@chem.bg.ac.rs

**Keywords:** grape pomace seed, skimmed goat’s milk, phenolics, milk protein, phenolic–protein interactions

## Abstract

The aim of this research was phenolics and protein characterization and antioxidant properties evaluation of skimmed thermally treated goat’s milk powder enriched with different concentration of grape pomace seed extract (SE). The dominant phenolics in SE were phenolic acids, flavan–3-ols and procyanidins. Different electrophoretic techniques together with UHPLC-MS/MS analysis revealed the presence of phenolics-protein interactions in the samples, mainly procyanidins with whey protein/caseins complexes. Addition of SE into thermally treated goat’s milk significantly improved antioxidant properties of goat’s milk such as TAC, FRP, DPPH^•^ and ABTS^•+^ scavenging activity. Gallic acid, catechin, and procyanidins mostly contributed to these activities. The schematic representation of phenolics–casein micelles interactions in thermally treated goat’s milk enriched with SE was given. The addition of SE into thermally treated goat’s milk can be a promising strategy in food waste recovery and to enhance the beneficial health effects of goat’s milk-based functional foods.

## 1. Introduction

Winemaking processes generate a considerable quantity of by-products (pomace, seed, skin and stem), which can be used as a rich source of highly valuable phenolic compounds (PCs) possessing broad range of biological activities, such as antioxidant, anti-carcinogenic, anti-inflammatory, and cardioprotective properties, etc. [1,2,3]. In addition to being motivated by environmental issues, the potential use of grape by-products gives the possibility of improving food quality and developing highly valuable ingredients [4,5]. It has been assumed that about 70% of the total phenolic compounds remain in grape pomace after processing, while the largest part of extractable phenolics originated from the seed which represents approximately 38–52% of solid by-products from the wine industry [4]. Grape seed extracts have exhibited good antioxidant activity by quenching free radicals, as previously estimated using in vitro antioxidant assays [3,6,7,8]. It gives them the potential to be used as a functional additive or synthetic antioxidant replacer in many food products [4].

As is well known, the most abundant phenolic compounds in grape seed are flavan–3-ols, procyanidins and phenolic acids [6,8]. They effectively reduce free radicals concentration, prevent their propagation, chelate some metal ions with their *o*-diphenol groups and thus prevent cell damage [2]. Therefore, numerous research studies are focused on extraction, valorization and application of grape pomace seed PCs [9,10], which represents one of the innovative strategies for waste recovery [11]. Until now, grape seed extracts are successfully incorporated in the several food products, such as cheese [12,13], yogurt [14], ice cream [15] or dry fermented sausages [16] contributing to the improvement of their antioxidant and nutritional properties, without negative organoleptic effects. However, the health benefits of procyanidin rich-grape seed extracts are limited due to their low recovery after digestion, provoked by their ability to polymerize and to react with digestive cocktail compounds [8]. However, their addition to the protein or carbohydrate rich food matrix has a protective effect and improves their recovery [8,17].

Milk is imposed as a promising carrier for PCs in the formulation of functional ingredients. It has been shown that a model system that includes a combination of whole and/or skim milk with phenolic-rich grape juice, preventing the degradation of high-valued phenolics, increases antioxidant activity and synergistically reduces the formation of lipid radicals [18,19,20]. It was also demonstrated that milk/milk proteins effectively encapsulate and interact with flavan–3-ols [21], improving their bioaccessibility [22] and transepithelial adsorption [23] of some specific catechin derivatives from tea, a rich source of flavan–3-ols. Moreover, the thermal treatment of milk leads to the “enrichment” of the casein micelle surface with the complex protein aggregates [24], which increases the probabilities of binding proteins with phenolics [25], and strengthen phenolics–caseins interactions [26].

Although the majority of these investigations were conducted on cow’s milk, a special attention has been recently paid on the study of goat’s milk. Until now, goat’s milk-based beverages with medicinal plant extracts have been successfully developed [27] and pollen-enriched skimmed goat’s milk powder [28]. To our knowledge, the potential synergistic effect between phenolic compounds and nutritionally valuable goat’s milk and the ability of the goat’s milk to retain antioxidant properties of grape pomace seed extracts has not yet been studied. Thus, the aim of this study was to prepare and to characterise phenolics and proteins of spray-dried skimmed goat’s milk powders enriched with different concentration of grape pomace seed extract and to evaluate their antioxidant activity. The results can be valuable for estimation of their possible use as functional ingredient in the formulation of healthier goat milk-based food products.

## 2. Materials and Methods

### 2.1. Preparation of Grape Pomace Seed Extract

Grape seed was separated from pomace of grape variety Prokupac, immediately after vinification process. Grape pomace was collected in winery (“Wine house Milinčić”), located in famous Serbian wine region Župa. Separated grape pomace seed was immediately grinded in a coffee grinder (Bosch MKM 6003 UC, BSH Hausgeräte GmbH, Munich, Germany) and PCs were extracted (1:10 *w/v*) by using 80% methanol (80/20 water:methanol), as described by Milinčić et al. [6]. After that, the grape pomace seed extract was evaporated (Heidolph, Laborota 4000, Schwabach, Germany) to dryness and reconstituted in Milli-Q water (SE) for further analysis and preparation of functional products.

#### UHPLC-Orbitrap MS^4^ Analysis of PCs in Aqueous Grape Pomace Seed Extract

Qualitative and quantitative analysis of PCs present in grape pomace seed extract were performed using an Accela Ultra high-performance liquid chromatography (UHPLC) system coupled to a linear ion trap-orbitrap mass spectrometer (LTQ OrbiTrap MS) equipped with a heated electrospray ionisation probe (HESI-II, ThermoFisher Scientific, Bremen, Germany) and Xcalibur software (version 2.1), as previously reported [6,8]. Quantification of all identified phenolic compounds was performed using available standards. The detection of phenolic compounds for which the standards were not been available was conducted based on their exact molecular masses and specific MS^4^ fragmentation. These compounds have been quantified and expressed as mg gallic acid; caffeic acid; catechin; quercetin–3-*O*-glucoside; or malvidin–3-*O*-glucoside per kg FW of seed, depending on the phenolic classes to which they belong.

### 2.2. Preparation of Milk and Milk/SE Powders

Goat’s milk samples were skimmed and thermally treated (90 °C, 10 min), as previously described by Pesic et al. [24]. After skimming, the skimmed goat’s milk (M) was separated and further used as a control for thermally treated goat’s milk (TM), in further electrophoretic characterization and antioxidant evaluation.

TM was mixed with three different quantities of aqueous seed extracts to enrich goat’s milk with different concentration of PCs. Based on the predetermined content of total phenolic compounds (TPC) in SE using the Folin–Cicolteu method [6], milk/SE mixtures were prepared in order to make the content of total phenolics as follow: 0.2 (TME1); 0.4 (TME2); and 0.6 (TME3) mg TPC per mL milk/SE mixture. Prepared samples, M, TM, TME and SE, were spray-dried using Buchi Mini B–290 spray dryer (Buchi Labortechnik AG, Flawil, Switzerland) with the same drying parameters as previously described by Kostić et al. 2021 [28]. The obtained powders were carefully packed in cuvettes, protected with a vapour-tight film and stored at −20 °C until further analysis.

#### 2.2.1. UHPLC-DAD MS/MS Analysis of PCs in Methanol Extracts of Powder Samples

Methanolic extracts of powder samples were prepared with the aim to characterize phenolics that contributed to the functionality of spray-dried powders. Approximately 1 g of powder sample was extracted with 10 mL of 80% methanol containing 0.1% HCl (acidified methanol) for 1 h. After that, samples were centrifuged at 3000× *g*, for 10 min, and collected supernatants were immediately analysed using a Dionex Ultimate 3000 UHPLC system equipped with a diode array detector (DAD) and TSQ Quantum Access Max triple-quadrupole mass spectrometer (Thermo Fisher Scientific, Basel, Switzerland), as previously reported by Pešić et al. [8]. Phenolics from the samples were quantified by direct comparison with commercially available standards (purchased of Sigma-Aldrich, Taufkirchen, Germany), using Xcalibur software (version 2.2.), and expressed as mg/g of spray-dried powder. A list of quantified phenolic compounds together with their equation parameters, limits of detection (LOD), limits of quantification (LOQ), correlation coefficient (r2) and linearity ranges are given in Table S1.

#### 2.2.2. Electrophoretic Analysis of Powder Samples

In this study, three electrophoretic techniques were used for protein profile characterization of prepared powder samples, as previously described by Pesic et al. [24]. SDS-PAGE in reducing (R) and non-reducing (NR) conditions were performed using separating (12.5% *w/v*; pH = 8.85) and stacking gels (5% *w/v*; pH = 6.8), as well as Tris-Glycine running buffer (0.05 M Tris (pH = 8.5), 0.19 M glycine, 0.1% *w/v* SDS). Native PAGE was carried out by using 7% (*w/v*) separating and 5% (*w/v*) stacking gels, as well as Tris-Glycine running buffer (0.025 M Tris (pH = 8.3), 0.19 M glycine).

Samples were prepared by dissolving 2 mg of spray-dried powder in appropriate sample buffers, which consisted of 0.055 M Tris-HCl (pH = 6.8), 2% (*w/v*) SDS, 7% (*v/v*) glycerol, 0.0025% (*w/v*) bromophenol blue and 5% β-mercaptoethanol for SDS-R-PAGE or without 5% β-mercaptoethanol for SDS-NR-PAGE; for native PAGE, sample buffer included 0.03 M Tris-HCl (pH = 8), 10% (*v/v*) glycerol and 0.0025% (*w/v*) Bromphenol blue. For all electrophoretic techniques, aliquots of 25 µL were loaded into the wells. After analysis was finished, gels were stained using Coomassie blue dye for 45 min, then destained, scanned and analyzed using SigmaGel software (SigmaGel software version 1.1, Jandal Scientific, San Rafael, CA, USA).

#### 2.2.3. TPC and Antioxidant Properties of Powder Samples

Spray-dried powder samples, 0.1; 0.5; and 1 g, were reconstituted in 100 mL Milli-Q water and adjusted to pH = 6.7. TPC of prepared samples were estimated using Folin–Ciocalteu method, as described by Kostić et al. [28]. Results were expressed as mg of GAE equivalents per 100 mL of sample (mg GAE/100 mL).

Antioxidant assays, such as total antioxidant capacity (in vitro phosphomolybdenum capacity) (TAC); ferric reducing power (FRP); ABTS^•+^ scavenging activity; and ferrous ion-chelating capacity (FCC), of prepared samples were evaluated as previously described by Pešić et al. [8] and Kostić et al. [28]. DPPH^•^ scavenging activity was evaluated using method developed by Oliveira et al. [29], with some modifications. Briefly, 105 μL of prepared samples were mixed with 840 μL of DPPH^•^ working solution and after incubation in the dark for 30 min, absorbance was measured at 515 nm (UV-1800, Shimadzu USA Manufacturing Inc., Canby, OR, USA). Obtained results for TAC, FRP, ABTS^•+^ and DPPH^•^ scavenging activity were expressed as µg of ascorbic acid equivalents per mL of sample (µg AAE/mL), while results for FCC was expressed as µg of EDTA equivalents per mL of sample (µg EDTA/mL).

### 2.3. Statistical Analysis

All analyses were conducted in triplicate. Data for antioxidant properties were analysed using general linear model procedure, considering treatments and suspension concentrations as fixed effects and replicate as a random effect. Significant differences between means were determined using post hoc, Tukey’s test (*p* < 0.05) (GraphPad Prism 6, San Diego, CA, USA). The Student’s *t*-test was used to evaluate the significantly differences between the means of individually phenolics, at *p* < 0.05 (StatSoft Co., Tulsa, OK, USA).

## 3. Results and Discussion

### 3.1. UHPLC-Orbitrap MS^4^ Characterisation of PCs in Aqueous Grape Pomace Seed Extract

Detailed identification, characterization and quantification of phenolic compounds of aqueous grape pomace seed extract, which was further used in the milk/SE formulation was performed using a UHPLC-Orbitrap MS^4^ analyzer (Table 1).

A total of 35 phenolic compounds were detected, which can be classified into several groups, as reported in the previous study [6]. Hydroxybenzoic acid and its derivatives, flavan–3-ols and procyandins, and hydroxycinnamic acid and its derivatives were the dominant classes of PCs in seed extracts, with a share of 50.58%; 29.42% and 17.13%, respectively. The dominant presence of phenolic acids, flavan–3-ols and procyanidins has been shown in some previous PCs characterization of seed from grape or pomace using chromatographic technique [6,7,8,30,31]. Gallic acid and its hexoside isomers, primarily gallic acid hexoside isomer 3 as well as ethyl gallate, were dominant among the individually detected phenolics, with content higher than 200 mg per kg FW of grape pomace seed. The high content of ethyl gallate (225.33 ± 9.35 mg/kg FW), which was also reported in a previous study by Milinčić et al. [6], can be attributed to the contact of the grape pomace seed and their dominant gallic acid derivatives with an alcoholic medium. Hydroxycinnamic derivatives such as caffeoyltartaric and cumaroyltartaric acids were found in significant amounts; however, they were reported in traces or absent in previously analyzed lyophilized grape seed extract [6]. This difference may be due to the vinification process, the presence of tartaric acid which easily interacts with phenolic acids, the intensive contact of the grape seed with the wine and extraction of the PCs immediately after separation from the wine and grape pomace without any additional treatment (drying or lyophilisation). Monomeric flavan–3-ols (catechin and epicatechin) and B type procyanidin isomers were predominantly present in Prokupac pomace seed, which is in line with previous characterizations of phenolics originating from the seed of this grape variety [6,8,31,32]. It has been observed that flavan–3-ols, procyanidins and phenolic acid derivatives had the highest influence on antioxidant activity of seed extracts, which was assessed using several in vitro assays [6].

Flavonol glycosides were present in traces (<1 mg/kg FW), while flavonol aglycones were not detected. Interestingly, some anthocyanin derivatives were registered (2.63% of the total phenolic content), although their complete absence in grape seed extracts is known. This may be due to the intensive contact of the grape seed with anthocyanins released from the skin during the vinification process their adsorption on the grape seed surface, primarily malvidin–3-*O*-glucoside which is dominant in the skin of Prokupac variety [8]. Also, the extraction of PCs from the seed immediately after separation from the grape pomace can also contributed to the detection of anthocyanin derivatives. Other detected anthocyanin derivatives were present in traces.

### 3.2. UHPLC-DAD MS/MS Analysis of PCs in Methanol Extracts of Powder Samples

The results of UHPLC-DAD MS/MS quantification of phenolic compounds in methanolic extracts of powder samples are listed in Table 2.

As can be seen, in reconstituted (80% acidified methanol) spray-dried SE and TME powders, 13 phenolic compounds were detected. This quantification also confirms the dominant presence of flavan–3-ol and phenolic acids (92.3% of total quantified phenolics), as previously reported [32,33]. Among individual phenolics, gallic acid (224.17 ± 3.94 mg/kg DW of powder) and catechin (518.28 ± 14.73 mg/kg DW of powder) were the most abundant; followed by quercetin and isorhamnetin aglycones, which were not previously confirmed by UHPLC-Orbitrap analysis of aqueous grape pomace seed extract. Detection of flavonol aglycones are probably a consequence of their hydrophobic nature, making them soluble in methanol/water solution. Other phenolic compounds in SE sample were present in traces.

Absence of phenolic compounds in methanolic extracts of M and TM powders were recorded. Taking this into account, phenolic compounds detected in samples of TME powders, originated exclusively from grape pomace seed extracts. The content of total PCs in the samples of TME powders increased with the amount of grape pomace seed extract added in the TME formulations, from 18.24 (TME1) to 71.30 (TME3) mg/kg DW of powder sample. The same increase of the content of dominant phenolics, such as catechin, gallic acid, catechin gallat and kaempferol, were observed in TME powder samples, while the content of caffeic acid did not change. The catechin content in TME powders were increased from 8.04 ± 0.14 (TME1) to 37.15 ± 1.60 (TME3) mg/kg DW of powder sample; and gallic acid content from 5.74 ± 0.40 (TME1) to 25.89 ± 1.12 (TME3) mg/kg DW of powder sample. Moreover, apart from catechin and catechin gallate, other flavan–3-ol derivatives present in the prepared mix of analytical phenolic standards were not detected in methanolic extracts of SE and TME powder samples. Interestingly, flavonol aglycones such as quercetin and isorhamnetin detected in methanolic extract of SE were not detected in acidified methanolic extracts of TME powder samples. This could be due to their strong hydrophobic interactions with proteins [17] that were not interrupted by extraction with acidified methanol.

### 3.3. Electrophoretic Analysis of M, TM and TME Powder Samples

**Figure 1:**
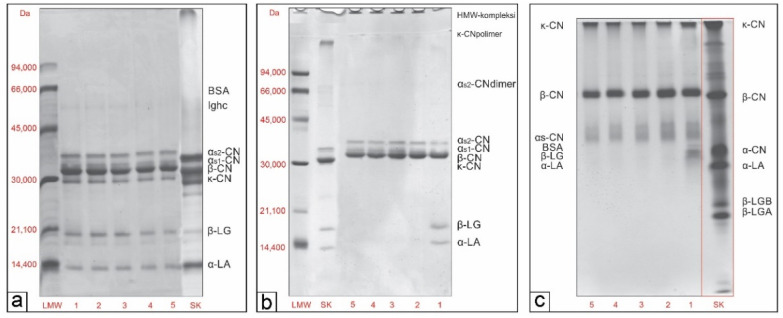
Electrophoretic patterns of M, TM and TME powders, analysed by SDS-PAGE in reducing conditions (SDS-R-PAGE) (**a**); SDS-PAGE in non-reducing conditions (SDS-NR-PAGE) (**b**); and native PAGE (Native-PAGE) (**c**). Lines: 1-Skimmed goat’s milk powder-M; 2-Skimmed thermally treated goat’s milk powder-TM; 3-Skimmed thermally treated goat’s milk/seed extract powder (0.2 mgTPC/mL)-TME1; 4-Skimmed thermally treated goat’s milk/seed extract powder (0.4 mgTPC/mL)-TME2; 5-Skimmed thermally treated goat’s milk/seed extract powder (0.6 mgTPC/mL)-TME3; Molecular weight standard (LMW); Bovine milk protein standard (SK). Abbreviations: bovine serum albumin (BSA); immunoglobulin hard chain (Ighc); αs2-casein (αs2-CN); αs1-casein (αs1-CN); β-casein (β-CN); κ-casein (κ-CN); β-lactoglobulins (β-LG); α-lactalbumin (α-LA).

Protein profiles of M, TM and TME powder samples were studied using three different electrophoretic techniques, SDS-PAGE under reducing (Figure 1a) and non-reducing (Figure 1b) conditions and native-PAGE (Figure 1c).

Protein detection was performed using bovine milk protein standards and available literature data [24]. As can be seen on SDS-R-PAGE patterns, the electrophoretic pathways of all samples (Figure 1a, lines 1–5) were identical with six dominant and well-known protein bands of goat’s milk that correspond to caseins and whey proteins. This means that the thermal treatment of goat’s milk (Figure 1a, line 2) or the addition of grape pomace seed extracts into the milk (Figure 1a, lines 3–5) did not changed the protein composition of skimmed goat’s milk (Figure 1a, lines 1). However, the intensity of the protein bands of α_S_-, β- and κ-casein decreased with increase amount of grape pomace seed extracts in the samples (TME1, TME2,TME3, Figure 1a, lines 3–5), in comparison to the same bands in TM sample (Figure 1a, line 2), as it is shown in Table 3. This can be explained by the presence of phenolics in the TME samples, leading to the decrease of protein content.

Generally, the average composition of α_S_-, β- and k-casein (calculated as % of total caseins) in all samples, determined by densitometric analysis of SDS-R-PAGE patterns, were 32.82 ± 1.37; 45.91 ± 1.11; 21.13 ± 1.37, respectively. These results are within the range of reported values for caprine caseins [34]. Moreover, the results for average α_S_-casein relative content were higher, while for β-casein were lower compared to the results previously reported by Pesic et al. [35]; however, both studies confirmed that β-casein is dominant in goat’s milk. The bands corresponding to BSA and immunoglobulins were of low intensity.

Differences between the samples can be observed on SDS-NR-PAGE patterns (Figure 1b), because the bonds between the formed complexes were not broken by the reducing agent. At the entrance to the upper gel, a band of high molecular weight (HMW) complexes can be noticed for all thermally treated milk samples with or without grape pomace seed extract (Figure 1b, lines 2–5). Intensity of bands corresponding to HMW complexes decreased in TME1, TME2 and TME3 (Figure 1b, lines 3–5) for 4.9%; 16.1% and 17.9%, respectively, compared to the same band in TM sample (Figure 1b, line 2) (Table 3). In the same samples (Figure 1b, lines 2–5), the complete absence of bands corresponding to β-LG, α-LA, κ-casein and α-S_2_-dimers can be observed probably due to their involvement in the formation of the heat-induced HMW complexes [24,36]. Moreover, Pesic et al. [24] showed that α-S_2_- and β-casein partially participate in the formation of heat-induced WP/CN complexes of goat’s milk, while k-casein participates dominantly with a share of over 70%. The same study confirmed the complete absence of soluble complexes in thermally treated goat’s milk.

Native-PAGE patterns confirmed the intensive denaturation and participation of whey proteins (β-LG and α-LA) and κ-casein in heat-induced complexes (Figure 1c, lines 2–5). Heat-induced whey protein aggregates have an increased affinity for binding phenolics and play a critical role in strengthening phenolics–casein interactions [25,26,37]. As can be observed (Figure 1c), the intensity of the bands corresponding to whey protein/casein (WP/CN) complexes of TME samples gradually increased with grape pomace seed extract amount in TME samples (TME1 < TME2 < TME3), in comparison to that of TM sample (Figure 1c, Table 3). These results indicated to the presence of interactions between phenolic compounds and heat-induced HMW complexes in TME samples that are not disturbed in non-dissociative and non-reducing conditions. The main phenolic compounds of grape pomace seed extract are gallic acid derivatives, flavan–3-ols and procyanidins. After preparation of powder samples, procyanidins as well as quercetin and isorhamnetin were not detected in acidified methanolic extracts indicated to their strong interactions with milk proteins. Several previous studies have shown that phenolic compounds of this type easily interact with milk proteins [38,39,40]. Changes in the intensity and mobility of these complex were not observed by SDS-NR-PAGE analysis of the same samples (Figure 1b, lines 2–5), because probably all hydrophobic and hydrogen phenolics-protein interactions were disrupted under the influence of strong dissociative agent. In addition, it can be observed that the intensity of the bands corresponding to β-casein and diffuse α_S_-casein, decreased with increasing amount of grape pomace seed extract in TME samples (Figure 1c, lines 3–5; Table 2), which is consistent with results of SDS-R-PAGE and SDS-NR-PAGE analysis. Obtaining results are in agreement with previous research by Kusuda et al. [41], who showed a significant effect of formed phenolics-protein complexes on the mobility and intensity of protein bands, using native-PAGE analysis. Moreover, they showed change in mobility of diffuse band of procyanidin-BSA complexes, while pentagalloylglucose (PPG)-BSA formed macromolecules had low mobility and remained at the entrance to the lower gel.

### 3.4. Total Phenolic Content and Antioxidant Properties

**Figure 2:**
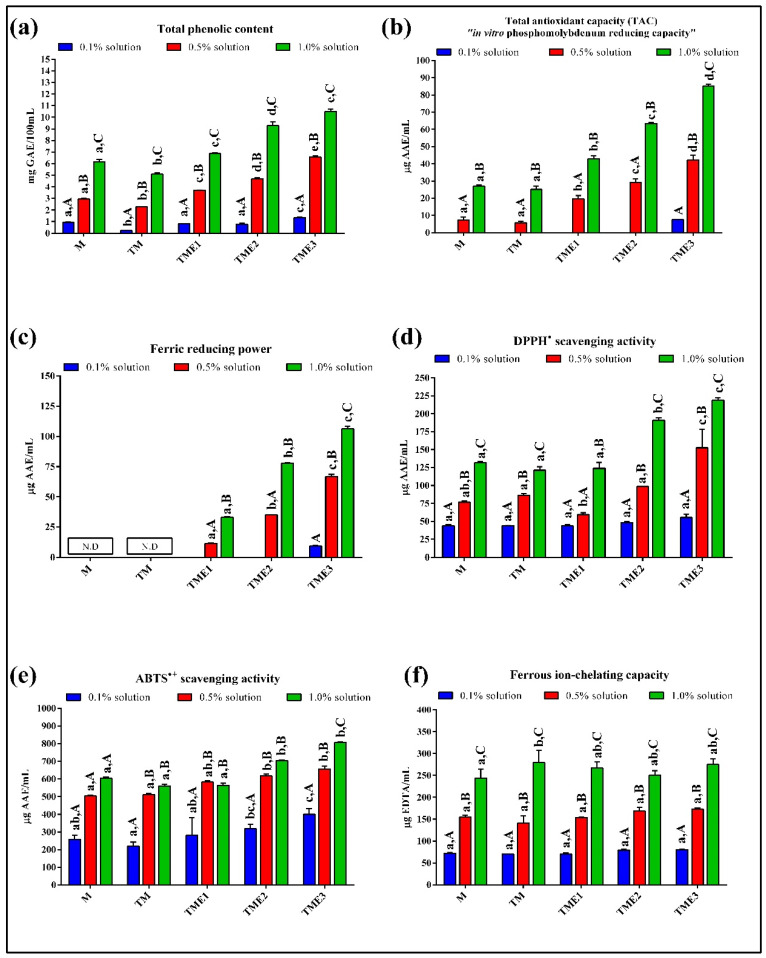
Total phenolic content (**a**) and antioxidant properties (**b**–**f**) of milk and milk/seed extract powders: (**b**) Phosphomolybdenium (TAC) assay; (**c**) Ferric reducing power; (**d**) DPPH^•^ scavenging activity; (**e**) ABTS^•+^ scavenging activity; and (**f**) Ferrous chelating capacity. The bars with (±) standard deviation represent mean values. Lowercase letters indicate comparisons of different samples for each concentration level; uppercase letters indicate comparisons within each sample among different concentration levels. Different letters indicate statistically significant differences according to Tukey’s test (*p* < 0.05). “N.D.”—not detected.

Numerous bioactive compounds have a protective effect against adverse by-products of oxidative metabolism, such as reactive oxygen species (ROS) and other free radicals [42,43]. These reactive species formed during normal physiological reactions in the organisms, are able to exist independently and stimulate oxidative chain reactions that can lead to serious problems at the cellular level [43]. The principle of neutralization of ROS and other free radicals by bioactive compounds is based on three different mechanisms of action: hydrogen atom transfer (HAT), single electron transfer (SET), and the ability to chelate transition metals [42,43,44,45], which are monitored through several antioxidant in vitro screening methods. In the case of complex food matrices, which contain more potential antioxidants, it is desirable to use a combination of several in vitro screening assays, in order to better interpret and understand the examined food system [43,44]. Therefore, in current study five in vitro antioxidant assays, such as TAC, ABTS^•+^/DPPH^•^ scavenging activity, FRP and FCC, as well as TPC, were used in this study to assess the antioxidant potential of M, TM and TME solutions at three concentration levels (Figure 2).

In the case of multicomponent samples, Folin–Ciocalteu (F-C) reagent can be reduced by many non-phenolic compounds that can give apparent higher concentrations of total phenolic compounds [42]. Therefore, this method measures the total reduction capacity of the samples [46]. Thus, although goat’s milk did not have phenolic compounds according to UHPLC-DAD analysis, TPC values obtained for these samples can be justified by the presence of a number of interfering substances such as proteins, amino acids and lactose that react with the F-C reagent [45]. With increasing M and TM concentration in the solutions, the reduction capacity substantially increased: 0.95; 2.94; and 6.15 mg GAE/100 mL of sample (M), or 0.23; 2.26; and 5.09 mg GAE/100 mL of sample (TM), for 0.1, 0.5, and 1% concentration levels, respectively (Figure 2a). Significantly lower TPC values for TM samples compared to M samples may be due to the protein modification after heat-treatment resulting in the lower number of reducing groups that were probably involved in the formation of HMW complexes already observed by SDS-NR-PAGE and native PAGE analysis.

The impact of skimmed and thermally treated goat’s milk on TPC values has been shown in other studies [27,28]. Samples of TM enriched with grape pomace seed extract had significantly higher TPC compared to that of TM samples. Phenolic compounds, mainly phenolic acids, flavan–3-ols and procyanidins of grape pomace seed extracts, were mostly contributed to TPC, since they were previously confirmed by UHPLC MS/MS analysis. The results of TPC values for TME samples showed a significant upward trend with increase of TME concentration (0.1; 0.5 and 1%), as well as with the increase of the amount of grape pomace seed extract in TME formulations. TPC values of TME samples in comparison with TM samples increased by 3.53; 3.32 and 5.85-fold for 0.1% solutions; 1.64; 2.07; 2.90-fold for 0.5% solutions; and 1.35; 1.82; 2.06 fold for 1% solutions of TME1; TME2; and TME3 samples, respectively.

As can be seen, the least increase of TPC values were in 1% TME samples, which may be due to the more intensive phenolics-protein interactions that interfered with their reducing capacity [17]. The TPC values obtained in this study are lower than some previously reported TPC values for goat’s milk beverages enriched with medicinal plant extracts [27], goat’s milk enriched with pollen [28], milk supplemented with grape polyphenol extract [20], fermented skim milk supplemented with grape pomace extract [47] or milk/grape juice beverages [19]. These differences may be due to applied methodology (most of these studies determined TPC after methanol extraction of PCs) and concentration of PCs added in functional products.

Total antioxidant capacity (TAC) was estimated based on the ability of M, TM and TME solutions (0.1; 0.5; and 1%) to reduce molybdenum ions from Mo (VI) to Mo (V), at acidic pH (Figure 2b). TAC of 0.1% solutions was recorded only for the TME3 sample, probably due to the sufficient phenolics concentration capable of performing metal ion reduction. Further increase of M, TM and TME concentration in the solutions (0.5 and 1%), showed Mo (VI) reducing ability; however, TAC values were significantly higher for TME samples in comparison to M and TM samples. The peptides and amino acids liberated by hydrolysis were probably the main contributors to the TAC values of M and TM, without statistically significant difference (*p* < 0.05) for both sample concentrations (0.5 and 1%). Nehir El et al. [48] were previously reported the ability of goat’s milk to reduce molybdenum ions. TAC values for both TME concentrations (0.5 and 1%) increased substantially with increasing phenolics concentration in TME1; TME2; and TME3 samples: 19.71; 29.16; 42.26 mg AAC/mL (0.5% solution) and 42.98; 63.44; and 85.07 mg AAC/mL (1% solution), respectively. TAC values of TME samples were probably contributed by free phenolics, protein-bound phenolic compounds liberated after hydrolysis of samples as well as by peptides and amino acids present in the samples.

FRP assay measures the ability of bioactive compounds to reduce ferric ions [42]. All M and TM samples (0.1; 0.5; and 1%) had no ferric reducing capacity, indicating poor ability of proteins to reduce ferric ions (Figure 2c). Low FRP values of raw, skimmed and thermally treated goat’s milk were previously reported by Abd El-Fattah et al. [49] and Kılıç Bayraktar et al. [20]. The addition of grape pomace seed extract to TM contributed to increase of FRP antioxidant activity. However, FRP values of 0.1% solutions were recorded only for the TME3 sample, which is in accordance with the TAC assay. Further increase of TME concentration in the solutions (0.5 and 1%), as well as phenolics concentration in the TME samples, significantly increased FRP values. The ability of TME samples to reduce ferric ions was probably the most contributed by free phenolics such as gallic acids and catechin. Similar, Kılıç Bayraktar et al. [20] demonstrated a significant influence of tea and grape polyphenols on FRP values of milk-polyphenol beverages.

The assessment of DPPH^•^ and ABTS^•+^ scavenging activity is based on the ability of M, TM and TME samples to donate hydrogen ions and neutralize free radicals. All samples at three concentration levels possessed the ability to neutralize DPPH^•^ and ABTS^•+^ radicals (Figure 2d,e). However, a lower quenching ability of DPPH^•^ than ABTS^•+^ was observed. This may be due to the hydrophobic nature of the DPPH^•^ and its ability to interact with lipophilic molecules [27,50]. DPPH^•^ scavenging activity of 0.1% solution were not significantly different among the samples (M; TM; TME), while ABTS^•+^ scavenging activity increased with increasing PCs content in TME samples. Further increase of sample concentration in solutions showed interesting results, i.e., the DPPH^•^ scavenging activity increased with increasing sample concentration (0.5 and 1%), while ABTS^•+^ scavenging activity was not significantly different for both concentration levels except for TME3 solutions. Good DPPH^•^ and ABTS^•+^ scavenging activity of M and TM samples was due to the presence of proteins, i.e., amino acids residues which are able to donate hydrogen ions [43]. Among individually milk proteins, the highest ABTS^• +^ scavenging activity was shown to have α-casein, followed by β-casein and β-Lg [51]. Moreover, thermal treatment of milk (TM) did not affect DPPH^•^ and ABTS^•+^ scavenging activity in comparison to raw milk (M), which is in accordance with the DPPH^•^ results reported by Abd El et al. [49]. Interestingly, DPPH^•^ scavenging activity of 0.5% TME1 sample was lower compared to M and TM samples which may be due to the phenolic–protein interactions that decrease their potential to donate hydrogen ions. It has been previously shown that caseins and whey proteins had ability to reduce the antioxidant activity of phenolics, due to phenolics-protein interactions [52,53]. However, TME2 and TME3 samples showed an increase in DPPH^•^ and ABTS^•+^ scavenging activity, to which increased phenolics content contributed. Due to methodological differences and the way in which DPPH^•^ and ABTS^•+^ results are expressed, comparison with other studies is difficult to conduct. However, previous studies have shown that ABTS^•+^ and DPPH^•^ scavenging capacity of goat’s milk significantly increased with the addition of phenolic rich plant extract [27] or pollen [28].

All samples showed good chelating properties as presented in Figure 2f, which can be associated with a high protein content [28]. Ferrous chelating capacity of 0.1 and 0.5% solutions were not significantly different (*p* < 0.05) between M, TM and TME samples. However, FFC of 1% TM solution significantly differed from 1% M solution, which may be due to the formation of heat-induced WP/CN complexes that probably improved FCC of TM sample. In summary, the addition of grape pomace seed extract to goat’s milk (TME) did not significantly alter the chelating properties of TM at all three concentration levels (0.1; 0.5; and 1%).

### 3.5. Model

**Figure 3:**
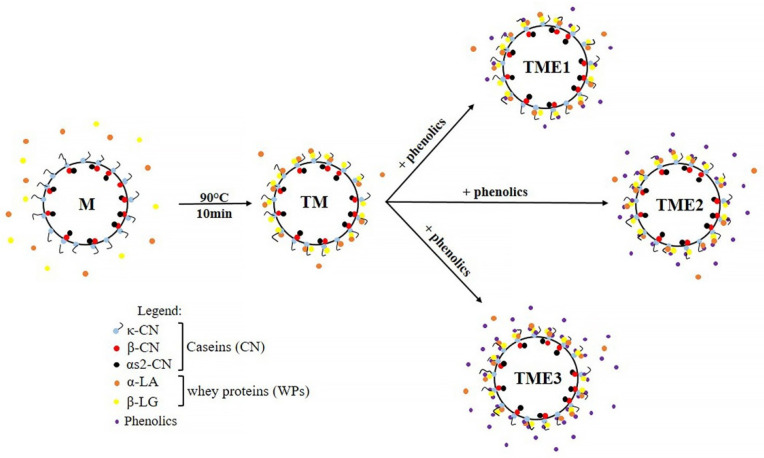
A schematic representation of the interactions between grape pomace seed phenolics at different concentration and casein micelles in thermally treated goat’s milk (90 °C, 10 min), at natural pH = 6.7. Abbreviations: skimmed goat’s milk (M); thermally treated goat’s milk (TM); skimmed thermally treated goat’s milk/seed extract powder (0.2 mgTPC/mL) (TME1); skimmed thermally treated goat’s milk/seed extract powder (0.4 mgTPC/mL) (TME2); skimmed thermally treated goat’s milk/seed extract powder (0.6 mgTPC/mL) (TME3).

Based on the obtained results in this study and previously presented model of the distribution of WPs in thermally treated goat’s milk [24], a new model of the distribution of phenolics in the thermally treated goat’s milk at natural pH = 6.7 can be proposed (Figure 3).

It was previously shown that thermal treatment of goat’s milk (90 °C, 10 min) induces a high percentage of denaturation of WPs and their aggregation with κ-casein and partially with α- and β-casein [24]. Participation of κ-; α- and β-casein in the formation of heat-induced complexes encourages better linkages and uniform distribution of micelle-bound complexes, which contribute to absence of free soluble complexes [24]. Previous reports by Rahimi Yazdi and Corredig [26] and Villalva et al. [37] have shown that this heat-induced WPs complexes increased and enhanced binding affinity of casein micelles to phenolic compounds.

Based on the results of electrophoretic and UHPLC MS/MS analysis, it can be assumed that grape pomace seed phenolics, due to low concentration in TME1 sample, interact with WP/CN complexes on the surface of casein micelles. However, increase in concentration of phenolics in TME2 and TME3 samples, leads to saturation of all binding sites on the surface of casein micelles (TME2), and penetration of phenolic compounds into the inner part of WP/CN complexes with greater access to hydrophobic regions of casein micelles (TME3). Binding affinities of phenolics to different parts of micelle-bound complexes depends on the phenolic compounds present in the grape pomace seed extracts. According to the report of Ye et al. [39], casein micelles are more likely to bind highly polymerized phenolics, while whey proteins tend to bind smaller phenolic molecules. Phenolics extracted from TME powders with acidified methanol and determined by UHPLC MS/MS analysis are probably free phenolics and phenolics bound by weaker bonds to the surface of casein micelles implicated that polymerized PCs such as procyanidins were bound with WP/CN complexes on the surface of casein micelles. Furthermore, quercetin and isorhamnetin were probably associated with denatured whey proteins present in the micellar phase of milk.

## 4. Conclusions

This study aims to characterize, valorize and apply the aqueous phenolics extract from grape pomace seed in the production of new goat’s milk-based food products or functional ingredients that can represent one of the innovative strategies in waste recovery and formulation of goat’s milk-based functional products. A total of 35 phenolic compounds were detected in the initial GE, among which phenolic acids and their derivatives, flavan–3-ols and procyandins were the dominant classes of PCs. In reconstituted spray-dried SE and TME powders, 13 phenolic compounds were detected, mainly flavan–3-ols and phenolic acids. The absence of other phenolic compounds could be due to their strong hydrophobic interactions with goat’s milk proteins. The interactions between phenolic compounds and goat’s milk proteins i.e., WP/CN complexes were confirmed by the different electrophoretic techniques (Native-PAGE; SDS-R-PAGE; SDS-NR-PAGE) and with different antioxidant tests.

The increase in extract amount in the prepared TME powders affected the increase in their antioxidant properties such as TAC, FRP, DPPH^•^ and ABTS^+•^ scavenging activity, which was mostly contributed by phenolic compounds (gallic acid, catechin and procyanidins). Also, the contribution of goat’s milk proteins on the antioxidant properties should not be neglected, primarily on ferrous chelating capacity, as well as their ability to interact and protect phenolic compounds as major antioxidants. Moreover, summarizing obtained results, a schematic representation of the interactions between phenolic compounds and casein micelles in thermally treated goat’s milk was proposed, which may contribute to future research in understanding phenolics-protein interactions and their effects on phenolics bioaccessibility during digestion. The complex and multicomponent nature of TME powders, as well as the positive synergistic contribution of phenolic compounds and goat’s milk proteins in improving antioxidant properties, potentially qualify TME powders as new functional food products or ingredients. Further research should be conducted to examine the techno-functional properties of the obtained TME powders.

## Figures and Tables

**Table 1:** The content of phenolic compounds (results are expressed as mg/kg FM) in aqueous grape pomace seed extract of Prokupac variety, determined using UHPLC-Orbitrap MS; retention time (*t*_R_), molecular formula, calculated/exact mass, mean mass accuracy (ppm), and major MS^2^ fragments.

Phenolic Compounds	*t*_R_, Min	Molecular Formula	Calculated Mass, [M–H]–/M^+^	Exact Mass, [M–H]–/M^+^	ppm	MS^2^ Fragments (% Base Peak)	Content (mg/kg FW)
	Hydroxybenzoic Acids and Derivatives						
**Gallic acid *^a^***	3.04	C_7_H_5_O_5_^−^	169.01425	169.01436	−0.11	125(100)	286.41 ± 8.28
**Gallic acid hexoside isomer 1 *^b^***	4.11	C_13_H_15_O_10_^−^	331.06707	331.06723	−0.16	271(40), 241(15), 211(20), 169(100), 125(10)	8.31 ± 0.26
**Dihydroxybenzoic acid hexoside *^b^***	4.30	C_13_H_15_O_9_^−^	315.07216	315.07251	−0.35	153(100), 152(50), 109(15), 108(10)	12.84 ± 0.99
**Gallic acid hexoside isomer 2 *^b^***	4.31	C_13_H_15_O_10_^−^	331.06707	331.06726	−0.19	294(10), 271(20), 169(100), 125(10)	66.65 ± 4.79
**Protocatechuic acid *^b^***	4.75	C_7_H_5_O_4_^−^	153.01933	153.01955	−0.22	109(100), 95(75), 79(20), 59(10)	27.92 ± 3.39
**Gallic acid hexoside isomer 3 *^b^***	4.76	C_13_H_15_O_10_^−^	331.06707	331.06592	1.15	169(100), 125(5)	222.47 ± 13.83
**Digalloyl hexoside *^b^***	5.08	C_20_H_19_O_14_^−^	483.07803	483.07764	0.39	331(20), 313(20), 271(100), 211(10), 169(10)	4.14 ± 0.30
**Methylgallate *^b^***	6.11	C_8_H_7_O_5_^−^	183.02990	183.03017	–0.27	168(100), 124(80)	0.64 ± 0.04
**Syringic acid hexoside *^b^***	6.21	C_15_H_19_O_10_^−^	359.09837	359.09837	0.00	197(100)	18.52 ± 0.35
**Ethylgallate *^b^***	7.16	C_9_H_9_O_5_^−^	197.04555	197.04530	0.25	169(100), 125(5)	225.33 ± 9.35
**Ellagic acid *^b^***	7.29	C_14_H_5_O_8_^−^	300.99899	300.99918	−0.19	284(40), 271(60), 257(100), 229(85), 185(40)	1.16 ± 0.09
**Σ**							874.39 (50.58)
	Hydroxycinnamic Acids and Derivatives						
**Caffeoyltartaric acid *^c^***	4.87	C_13_H_11_O_9_^−^	311.04031	311.04141	−1.10	179(40), 177(15), 149(100)	193.78 ± 6.42
**Caffeic acid *^a^***	5.38	C_9_H_7_O_4_^−^	179.03498	179.03545	−0.47	135(100)	32.82 ± 1.96
**Coumaroyltartaric acid *^c^***	5.60	C_13_H_11_O_8_^−^	295.04594	295.04623	−0.29	163(100), 149(10), 119(5)	72.63 ± 2.46
**Σ**							299.24 (17.13)
	Flavan–3-ols and Procyanidins						
**B type procyanidin trimer isomer 1 *^d^***	4.69	C_45_H_37_O_18_^-^	865.19854	865.20264	−4.10	695(100), 577(60), 425(30), 407(30), 287(30)	13.07 ± 0.24
**B type procyanidin dimer isomer 1 *^d^***	5.47	C_30_H_25_O_12_^−^	577.13515	577.13318	1.97	559(10), 451(30), 425(100), 407(40), 289(20), 287(10)	61.39 ± 3.31
**B type procyanidin dimer isomer 2 *^d^***	5.72	C_30_H_25_O_12_^−^	577.13515	577.13531	−0.16	559(5), 451(20), 425(100), 407(35), 289(20), 287(10)	46.18 ± 2.74
**B type procyanidin trimer isomer 2 *^d^***	5.73	C_45_H_37_O_18_^−^	865.19854	865.20087	−2.33	695(100), 577(80), 425(30), 407(40), 287(35)	34.51 ± 1.04
**B type procyanidin dimer isomer 3 *^d^***	6.02	C_30_H_25_O_12_^−^	577.13515	577.13379	1.36	559(10), 451(20), 425(100), 407(40), 289(20), 287(10)	91.54 ± 3.15
**Catechin *^a^***	6.17	C_15_H_13_O_6_^−^	289.07176	289.07089	0.87	271(5), 245(100), 205(40), 179(15), 125(5)	83.45 ± 3.48
**B type procyanidin dimer gallate isomer 1 *^d^***	6.23	C_37_H_29_O_16_^−^	729.14611	729.14734	−1.23	577(90), 559(80), 425(20), 407(100), 289(20)	14.82 ± 0.65
**B type procyanidin dimer gallate isomer 2 *^d^***	6.44	C_37_H_29_O_16_^−^	729.14611	729.14728	−1.17	577(50), 559(60), 425(10), 407(100), 289(20)	61.99 ± 1.65
**Epicatechin *^d^***	6.54	C_15_H_13_O_6_^−^	289.07176	289.07068	1.08	271(5), 245(100), 205(40), 179(15), 125(5)	90.58 ± 2.51
**B type procyanidin dimer digallate *^d^***	6.80	C_44_H_33_O_20_^−^	881.15707	881.15723	−0.16	729(100), 711(30), 577(10), 559(20), 407(30)	0.93 ± 0.07
**Catechin gallate *^d^***	7.09	C_22_H_17_O_10_^−^	441.08272	441.08218	0.54	331(10), 289(100), 271(10), 169(25)	10.10 ± 0.57
**Σ**							508.56 (29.42)
	Flavonol Aglycones and Glycosides						
**Dihydro-syringetin–3-*O*-hexoside *^e^***	5.54	C_23_H_25_O_13_^−^	509.13006	509.12967	0.39	491(10), 461(30), 355(40), 347(65), 329(100)	0.54 ± 0.06
**Quercetin–3-*O*-glucoside *^a^***	7.06	C_21_H_19_O_12_^−^	463.08820	463.08786	0.34	301(100), 300(30)	0.37 ± 0.04
**Σ**							0.915(0.05)
	Anthocyanins						
**Delphinidin–3-*O*-glucoside *^a^***	4.85	C_21_H_21_O_12_^+^	465.10275	465.10294	−0.41	304(15), 303(100)	1.29 ± 0.09
**Petunidin–3-*O*-hexoside *^f^***	5.27	C_22_H_23_O_12_^+^	479.11840	479.11884	−0.92	318(10), 317(100)	1.48 ± 0.10
**Peonidin–3-*O*-glucoside *^a^***	5.53	C_22_H_23_O_11_^+^	463.12349	463.12366	−0.37	302(10), 301(100)	2.56 ± 0.22
**Malvidin–3-*O*-glucoside *^a^***	5.59	C_23_H_25_O_12_^+^	493.13405	493.13394	0.22	332(10), 331(100)	28.53 ± 3.32
**Peonidin–3-*O*-(6″-acetyl)hexoside *^f^***	6.42	C_24_H_25_O_12_^+^	505.13405	505.13351	0.54	302(10), 301(100)	1.32 ± 0.12
**Malvidin–3-*O*-(6″-acetyl)hexoside *^f^***	6.48	C_25_H_27_O_13_^+^	535.14462	535.14398	0.64	332(10), 331(100)	6.14 ± 0.55
**Peonidin–3-*O*-(6″-*p*-coumaroyl)hexoside *^f^***	7.12	C_31_H_29_O_13_^+^	609.16027	609.16077	−0.50	302(10), 301(100)	1.44 ± 0.16
**Malvidin–3-*O*-(6″-*p*-coumaroyl)hexoside *^f^***	7.18	C_32_H_31_O_14_^+^	639.17083	639.17163	−0.80	332(10), 331(100)	2.77 ± 0.42
**Σ**							45.54 (2.63)
**Σ**							1728.64

Content of phenolics are presented as mean values ± standard deviations (*n* = 3); Values in parenthesis represent relative amount of phenolic class in seed extract; *^a^* Compounds quantified using available standards; Compounds that were quantified and expressed as equivalents of gallic acid *^b^*; caffeic acid *^c^*; catechin *^d^*; quercetin–3-*O*-glucoside *^e^*; malvidin–3-*O*-glucoside *^f^*.

**Table 2:** The content of phenolic compounds in methanolic extracts of M, TM, SE and TME powders (results are expressed as mg/kg SM of powders), determined using UHPLC-DAD MS/MS.

Samples	M	TM	SE	TME1	TME2	TME3
Compounds (mg/kg DW of Powders)						
Fenolic Acid and its Derivatives						
Gallic acid	n.d.	n.d.	224.17 ± 3.94 ^a^	5.74 ± 0.40 ^b^	12.64 ± 0.30 ^c^	25.89 ± 1.12 ^d^
Protocatechuic acid	n.d.	n.d.	2.43 ± 0.08	n.d.	n.d	n.d
Syringic acid	n.d.	n.d.	1.84 ± 0.07	n.d.	n.d	n.d
Caffeic acid	n.d.	n.d.	2.24 ± 0.20 ^a^	1.26 ± 0.06 ^b^	1.31 ± 0.09 ^b^	1.25 ± 0.10 ^b^
Flavan–3-ols and its Derivatives						
Catechin	n.d.	n.d.	518.28 ± 14.73 ^a^	8.04 ± 0.14 ^b^	18.90 ± 0.92 ^c^	37.15 ± 1.60 ^d^
Catechin gallat	n.d.	n.d.	8.36 ± 0.13 ^a^	0.83 ± 0.02 ^b^	1.52 ± 0.08 ^c^	2.72 ± 0.14 ^d^
Gallocatechin	n.d.	n.d.	n.d.	n.d.	n.d.	n.d.
Epigallocatechin	n.d.	n.d.	n.d.	n.d.	n.d.	n.d.
Epigallocatechin gallat	n.d.	n.d.	n.d.	n.d.	n.d.	n.d.
Flavanol Aglycones i Glycosides						
Quercetin	n.d.	n.d.	32.66 ± 2.10	n.d.	n.d	n.d
Quercetin–3-glucoside	n.d.	n.d.	1.51 ± 0.07 ^a^	0.51 ± 0.02 ^b^	0.89 ± 0.05 ^c^	1.41 ± 0.06 ^a^
Rutin	n.d.	n.d.	0.30 ± 0.02 ^a^	0.36 ± 0.02 ^b^	0.37 ± 0.03 ^b^	0.46 ± 0.04 ^c^
Isorhramnetin	n.d.	n.d.	17.31 ± 0.87	n.d.	n.d	n.d
Isorhramnetin–3-*O*-glucoside	n.d.	n.d.	n.d.	0.15 ± 0.01 ^a^	0.26 ± 0.02 ^b^	0.60 ± 0.03 ^c^
Kaempferol	n.d.	n.d.	7.90 ± 0.34 ^a^	1.20 ± 0.02 ^b^	1.41 ± 0.04 ^c^	1.67 ± 0.01 ^d^
Other Detected Phenolics						
Apigenin–7-glucoside	n.d.	n.d.	n.d.	0.16 ± 0.01 ^a^	n.d	0.18 ± 0.02 ^a^
Naringenin	n.d.	n.d.	0.81 ± 0.04	n.d.	n.d	n.d
Aesculetin	n.d.	n.d.	2.79 ± 0.20	n.d.	n.d	n.d
Σ Σ	/	/	820.59	18.24	37.30	71.35

Values are presented as mean values ± standard deviations (*n* = 3); Different letters in the same order denote a significant difference according to *t*-test, *p* < 0.05. “n.d.”—compound not detected; Abbreviations: methanolic extracts of spray dried milk (M); thermally treated milk (TM); grape pomace seed extract (SE); thermally treated milk/seed extract (TME).

**Table 3:** The change (%) of caseins and HMW complexes content in TME samples in relation to the same band in TM sample, determined by different electrophoretic techniques.

Samples	TM	TME1	TME2	TME3
	*SDS-R-PAGE*			
αS2-CN	100 ^a^	95.1 ± 2.6 ^b^	90.8 ± 3.8 ^bc^	88.3 ± 2.2 ^c^
β-CN	100 ^a^	87.1 ± 0.9 ^b^	85.8 ± 1.2 ^b^	82.9 ± 0.6 ^c^
κ-CN	100 ^a^	89.3 ± 2.6 ^b^	88.7 ± 3.9 ^b^	77.0 ± 2.6 ^c^
	*SDS-NR-PAGE*			
HMW complexes	100 ^a^	93.7 ± 3.6 ^b^	82.5 ± 3.2 ^c^	80.8 ± 4.2 ^c^
	*Native-PAGE*			
WPs/CN complexes	100 ^c^	103.9 ± 3.0 ^bc^	110.7 ± 5.4 ^b^	131.4 ± 8.6 ^a^
β-CN	100 ^a^	89.8 ± 1.2 ^b^	84.6 ± 1.6 ^c^	80.6 ± 1.4 ^d^

Values are presented as mean values ± standard deviations (*n* = 3). Different letters in the same order denote a significant difference according to *t*-test, *p* < 0.05. Abbreviations: Skimmed thermally treated goat’s milk powder (TM); Skimmed thermally treated goat’s milk/seed extract powder (0.2 mgTPC/mL) (TME1); Skimmed thermally treated goat’s milk/seed extract powder (0.4 mgTPC/mL) (TME2); Skimmed thermally treated goat’s milk/seed extract powder (0.6 mgTPC/mL) (TME3).

## Data Availability

No new data were created or analyzed in this study. Data sharing is not applicable to this article.

## Supplementary Materials

The following are available online at https://www.mdpi.com/article/10.3390/biom11070965/s1, Table S1: Determination characteristics of phenolic compounds and analytical performance of the method using UHPLC- MS/MS Orbitrap.

## References

[1] Lei C., Tang X., Li H., Chen H., Yu S. (2020). Molecular hybridization of grape seed extract: Synthesis, structural characterization and anti-proliferative activity in vitro. Food Res. Int..

[2] Unusan N. (2020). Proanthocyanidins in grape seeds: An updated review of their health benefits and potential uses in the food industry. J. Funct. Foods.

[3] Yu J., Ahmedna M. (2013). Functional components of grape pomace: Their composition, biological properties and potential applications. Int. J. Food Sci. Technol..

[4] Beres C., Costa G.N., Cabezudo I., da Silva-James N.K., Teles A.S., Cruz A.P., Mellinger-Silva C., Tonon R.V., Cabral L.M., Freitas S.P. (2017). Towards integral utilization of grape pomace from winemaking process: A review. Waste Manag..

[5] Muhlack R.A., Potumarthi R., Jeffery D.W. (2018). Sustainable wineries through waste valorisation: A review of grape marc utilisation for value-added products. Waste Manag..

[6] Milinčić D.D., Stanisavljević N.S., Kostić A.Ž., Bajić S.S., Kojić M.O., Gašić U.M., Barać M.B., Stanojević S.P., Tešić Ž.L., Pešić M.B. (2021). Phenolic compounds and biopotential of grape pomace extracts from Prokupac red grape variety. LWT.

[7] Rockenbach I.I., Jungfer E., Ritter C., Santiago-Schübel B., Thiele B., Fett R., Galensa R. (2012). Characterization of flavan-3-ols in seeds of grape pomace by CE, HPLC-DAD-MSn and LC-ESI-FTICR-MS. Food Res. Int..

[8] Pešić M.B., Milinčić D., Kostić A.Ž., Stanisavljević N.S., Vukotic G., Kojic M., Gašić U., Barać M.B., Stanojevic S., Popović D.A. (2019). In vitro digestion of meat- and cereal-based food matrix enriched with grape extracts: How are polyphenol composition, bioaccessibility and antioxidant activity affected?. Food Chem..

[9] Fontana A.R., Antoniolli A., Bottini R. (2013). Grape Pomace as a Sustainable Source of Bioactive Compounds: Extraction, Characterization, and Biotechnological Applications of Phenolics. J. Agric. Food Chem..

[10] García-Lomillo J., González-SanJosé M.L. (2017). Applications of Wine Pomace in the Food Industry: Approaches and Functions. Compr. Rev. Food Sci. Food Saf..

[11] López-Belchí M., Caamaño E., Pascual G., Noriega F., Fierro-Morales P., Romero-Román M., Jara P., Schoebitz M., Serra I., Moreno D. (2021). Spray-Dried Formulations Rich in Malvidin from Tintorera Grape Wastes: Characterization, Stability, and Storage. Processes.

[12] Deolindo C.T.P., Monteiro P.I., Santos J.S., Cruz A.G., da Silva M.C., Granato D. (2019). Phenolic-rich Petit Suisse cheese manufactured with organic Bordeaux grape juice, skin, and seed extract: Technological, sensory, and functional properties. LWT.

[13] Da Silva D.F., Matumoto-Pintro P.T., Bazinet L., Couillard C., Britten M. (2015). Effect of commercial grape extracts on the cheese-making properties of milk. J. Dairy Sci..

[14] Chouchouli V., Kalogeropoulos N., Konteles S.J., Karvela E., Makris D.P., Karathanos V.T. (2013). Fortification of yoghurts with grape (*Vitis vinifera*) seed extracts. LWT.

[15] Sagdic O., Ozturk I., Cankurt H., Tornuk F. (2012). Interaction between Some Phenolic Compounds and Probiotic Bacterium in Functional Ice Cream Production. Food Bioprocess. Technol..

[16] Ribas-Agustí A., Gratacós-Cubarsí M., Sárraga C., Guàrdia M.D., García-Regueiro J.-A., Castellari M. (2014). Stability of phenolic compounds in dry fermented sausages added with cocoa and grape seed extracts. LWT.

[17] Jakobek L. (2015). Interactions of polyphenols with carbohydrates, lipids and proteins. Food Chem..

[18] Lamothe S., Guérette C., Dion F., Sabik H., Britten M. (2019). Antioxidant activity of milk and polyphenol-rich beverages during simulated gastrointestinal digestion of linseed oil emulsions. Food Res. Int..

[19] He Z., Tao Y., Zeng M., Zhang S., Tao G., Qin F., Chen J. (2016). High pressure homogenization processing, thermal treatment and milk matrix affect in vitro bioaccessibility of phenolics in apple, grape and orange juice to different extents. Food Chem..

[20] Bayraktar M.K., Harbourne N., Fagan C.C. (2019). Impact of heat treatment and acid gelation on polyphenol enriched milk samples. LWT.

[21] Lorenz M., Jochmann N., Von Krosigk A., Martus P., Baumann G., Stangl K., Stangl V. (2006). Addition of milk prevents vascular protective effects of tea. Eur. Heart J..

[22] Moser S., Chegeni M., Jones O.G., Liceaga A., Ferruzzi M.G. (2014). The effect of milk proteins on the bioaccessibility of green tea flavan-3-ols. Food Res. Int..

[23] Xie Y., Kosińska A., Xu H., Andlauer W. (2013). Milk enhances intestinal absorption of green tea catechins in in vitro digestion/Caco-2 cells model. Food Res. Int..

[24] Pesic M.B., Barac M., Stanojevic S., Ristic N.M., Macej O.D., Vrvic M. (2012). Heat induced casein–whey protein interactions at natural pH of milk: A comparison between caprine and bovine milk. Small Rumin. Res..

[25] Taterka H., Castillo M. (2015). The effect of whey protein denaturation on light backscatter and particle size of the casein micelle as a function of pH and heat-treatment temperature. Int. Dairy J..

[26] Yazdi S.R., Corredig M. (2012). Heating of milk alters the binding of curcumin to casein micelles. A fluorescence spectroscopy study. Food Chem..

[27] Verruck S., Dantas A., Prudencio E.S. (2019). Functionality of the components from goat’s milk, recent advances for functional dairy products development and its implications on human health. J. Funct. Foods.

[28] Komes D., Busic A., Belščak-Cvitanović A., Brnčić M., Bosiljkov T., Vojvodic A., Dujmic F. (2017). Novel Approach to the Development of Functional Goat’s Milk-Based Beverages Using Medicinal Plant Extracts in Combination with High Intensity Ultrasound Treatment. Food Technol. Biotechnol..

[29] Kostić A.Ž., Milinčić D.D., Stanisavljević N.S., Gašić U.M., Lević S., Kojić M.O., Tešić Ž.L., Nedović V., Barać M.B., Pešić M.B. (2021). Polyphenol bioaccessibility and antioxidant properties of in vitro digested spray-dried thermally-treated skimmed goat milk enriched with pollen. Food Chem..

[30] Oliveira A., Silva L., Ferreres F., de Pinho P.G., Valentão P., Silva B.M., Pereira J.A., Andrade P.B. (2010). Chemical Assessment and in Vitro Antioxidant Capacity of Ficus carica Latex. J. Agric. Food Chem..

[31] Peixoto C.M., Dias M.I., Alves M.J., Calhelha R.C., Barros L., Pinho S.P., Ferreira I.C. (2018). Grape pomace as a source of phenolic compounds and diverse bioactive properties. Food Chem..

[32] Zdunić G., Gođevac D., Šavikin K., Krivokuća D., Mihailović M., Pržić Z., Marković N. (2019). Grape Seed Polyphenols and Fatty Acids of Autochthonous Prokupac Vine Variety from Serbia. Chem. Biodivers..

[33] Godjevac D., Tesevic V., Velickovic M., Vujisic L., Vajs V., Milosavljevic S. (2010). Polyphenolic compounds in seeds from some grape cultivars grown in Serbia. J. Serb. Chem. Soc..

[34] Kammerer D., Claus A., Carle R., Schieber A. (2004). Polyphenol Screening of Pomace from Red and White Grape Varieties (*Vitis vinifera* L.) by HPLC-DAD-MS/MS. J. Agric. Food Chem..

[35] Pesic M.B., Barac M., Stanojevic S., Vrvic M. (2014). Effect of pH on heat-induced casein-whey protein interactions: A comparison between caprine milk and bovine milk. Int. Dairy J..

[36] Dalgleish D.G., Corredig M. (2012). The Structure of the Casein Micelle of Milk and Its Changes during Processing. Annu. Rev. Food Sci. Technol..

[37] Villalva M., Jaime L., Arranz E., Zhao Z., Corredig M., Reglero G., Santoyo S. (2020). Nanoemulsions and acidified milk gels as a strategy for improving stability and antioxidant activity of yarrow phenolic compounds after gastrointestinal digestion. Food Res. Int..

[38] Prigent S., Voragen A., Van Koningsveld G., Baron A., Renard C.M., Gruppen H. (2009). Interactions between globular proteins and procyanidins of different degrees of polymerization. J. Dairy Sci..

[39] Ye J.-H., Fan F., Xu X., Liang Y. (2013). Interactions of black and green tea polyphenols with whole milk. Food Res. Int..

[40] Kanakis C., Hasni I., Bourassa P., Tarantilis P., Polissiou M., Tajmir-Riahi H.-A. (2011). Milk β-lactoglobulin complexes with tea polyphenols. Food Chem..

[41] Kusuda M., Hatano T., Yoshida T. (2006). Water-Soluble Complexes Formed by Natural Polyphenols and Bovine Serum Albumin: Evidence from Gel Electrophoresis. Biosci. Biotechnol. Biochem..

[42] Gülçin I. (2012). Antioxidant activity of food constituents: An overview. Arch. Toxicol..

[43] Power O., Jakeman P., Fitzgerald R.J. (2012). Antioxidative peptides: Enzymatic production, in vitro and in vivo antioxidant activity and potential applications of milk-derived antioxidative peptides. Amino Acids.

[44] Granato D., Shahidi F., Wrolstad R., Kilmartin P., Melton L.D., Hidalgo F.J., Miyashita K., van Camp J., Alasalvar C., Ismail A. (2018). Antioxidant activity, total phenolics and flavonoids contents: Should we ban in vitro screening methods?. Food Chem..

[45] Prior R.L., Wu X., Schaich K. (2005). Standardized Methods for the Determination of Antioxidant Capacity and Phenolics in Foods and Dietary Supplements. J. Agric. Food Chem..

[46] Huang D., Ou B., Prior R.L. (2005). The Chemistry behind Antioxidant Capacity Assays. J. Agric. Food Chem..

[47] Azevedo P.O.D.S.D., Aliakbarian B., Casazza A.A., Leblanc J.G., Perego P., Oliveira R.P.D.S. (2018). Production of fermented skim milk supplemented with different grape pomace extracts: Effect on viability and acidification performance of probiotic cultures. PharmaNutrition.

[48] El S.N., Karakaya S., Simsek S., Dupont D., Menfaatli E., Eker A.T. (2015). In vitro digestibility of goat milk and kefir with a new standardised static digestion method (INFOGEST cost action) and bioactivities of the resultant peptides. Food Funct..

[49] El-Fattah A.A., Azzam M., Elkashef H., Elhadydy A. (2019). Antioxidant Properties of Milk: Effect of Milk Species, Milk Fractions and Heat Treatments. Int. J. Dairy Sci..

[50] Serpen A., Gökmen V., Fogliano V. (2012). Total antioxidant capacities of raw and cooked meats. Meat Sci..

[51] Kim Y., Kim J.W., Cheon S., Nam M.S., Kim K.K. (2019). Alpha-Casein and Beta-Lactoglobulin from Cow Milk Exhibit Antioxidant Activity: A Plausible Link to Antiaging Effects. J. Food Sci..

[52] Gallo M., Vinci G., Graziani G., De Simone C., Ferranti P. (2013). The interaction of cocoa polyphenols with milk proteins studied by proteomic techniques. Food Res. Int..

[53] Li T., Li X., Dai T., Hu P., Niu X., Liu C., Chen J. (2020). Binding mechanism and antioxidant capacity of selected phenolic acid—β-casein complexes. Food Res. Int..

