# Supplementary material for: Skimmed Goat’s Milk Powder Enriched with Grape Pomace Seed Extract: Phenolics and Protein Characterization and Antioxidant Properties
Source: Biomolecules. 2021 Jun 30;11(7):965. doi: 10.3390/biom11070965 (PMC8301875; doi:10.3390/biom11070965)
Supplement: Supplementary file 1 [file biomolecules-11-00965-s001.zip › biomolecules-1261044-supplementary.pdf]

Skimmed goat milk powder enriched with grape pomace seed extract: Phenolics and protein characterization and antioxidant properties

Danijel D. Milinčić[1], Aleksandar Ž. Kostić[1], Uroš M. Gašić[2], Steva Lević[1], Slađana P. Stanojević[1], Miroljub B. Barać[1], Živoslav Lj. Tešić[3], Viktor Nedović[1], and Mirjana B. Pešić[1,*]

Table S1. Determination characteristics of phenolic compounds and analytical performance of the method using UHPLC- MS/MS Orbitrap

Compound name	Linear regression ($y=a+b*x$)	r^2	LOD (mg/L)	LOQ (mg/L)	Linear range (mg/L)
**Gallic acid**	y = -2.08E+7 + 3.86E+7*x	0.9908	0.17	0.56	0.50 - 2.00
**Caffeic acid**	y = - 1.30E+6 + 2.43E+7*x	0.9939	0.17	0.58	0.01 - 1.90
**Catechin**	y = - 3.56E+4+ 3.60E+5*x	0.9977	0.08	0.26	0.15 - 1.50
**Quercetin-3-O-glucoside**	y = 7.13E+5 + 3.33E+7*x	0.9986	0.08	0.28	0.01 - 2.00
**Malvidin-3-*O*-glucoside**	y = - 1.04E+4 + 6.94E+5*x	0.9931	0.09	0.31	0.05 - 1.00
**Delphinidin 3-O-glucoside**	y = - 1.73E+4 + 4.26E+5*x	0.9988	0.04	0.13	0.05 - 1.00
**Peonidin 3-O-glucoside**	y = - 7.44E+3 + 2.86E+5*x	0.9916	0.10	0.33	0.05 - 1.00
